# Supplementary material for: Maternal One-Carbon Metabolism during the Periconceptional Period and Human Foetal Brain Growth: A Systematic Review
Source: Genes (Basel). 2021 Oct 17;12(10):1634. doi: 10.3390/genes12101634 (PMC8535925; doi:10.3390/genes12101634)
Supplement: Supplementary file 1 [file genes-12-01634-s001.zip › genes-1399885-supplementary.pdf]

Table S1. Main terms used for the literature search on Embase.com

One carbon metabolism	AND	Nervous system development	AND	Pregnancy
Carbon metabolism		Brain size		Developmental biology
Folic acid		Brain growth		Embryo
Homocysteine		Neurulation		Fetus
Cyanocobalamin		Synaptogenesis		Prenatal period
B12 deficiency		Brain development		Prenatal exposure
Methionine		Neural maturation		Gestation period

Table S2. ErasmusAGE quality scoring system. This quality score can be used to assess the quality of studies included in systematic reviews and meta-analyses and is applicable to both interventional and observational studies. The score was designed based on previously published scoring systems (Carter et al, 2010 and the Quality Assessment Tool for Quantitative Studies*)*. The quality score is composed of 5 items, and each item is allocated 0, 1 or 2 points. This allows a total score between 0 and 10 points, 10 representing the highest quality.

Study design	0 for studies with cross-sectional data collection<br>1 for studies with longitudinal data collection (both retrospective and prospective)<br>2 for intervention studies
Study size	0 small population for analysis (N = 20-199)<br>1 intermediate population for analysis (N = 200-2999)<br>2 large population for analysis (N > 3000)
Exposure	*Observational studies*<br>0 if the study used no appropriate exposure measurement method or if not reported<br>1 if the study used moderate quality exposure measurement methods<br>2 if the study used adequate exposure measurement methods<br><br>*Intervention studies*<br>0 if the intervention was not described or not blinded<br>1 if the intervention was adequately single blinded.<br>2 if the intervention was adequately double-blinded.
Outcome	0 if the study used no appropriate outcome measurement method or if not reported<br>1 if the study used moderate quality outcome measurement methods (one fetal outcome measure)<br>2 if the study used adequate outcome measurement methods (multiple fetal outcome measures)
Adjustments	0 if findings are not controlled for at least key confounders<br>1 if findings are controlled for key confounders<br>2 if findings are additionally controlled for additional covariates or when an intervention is adequately randomized

Table S3. Main characteristics of the 26 included studies.

Author (year)	Country	Study period	Study design	Study population	Sample size	Exposures	Outcome(s)	Quality score
Bergen *et al.* (2016)	Netherlands	2001-2005	Cohort study	Healthy pregnant women	5890	Plasma tHcy, serum vitamin B_{12}, plasma folate	Foetal HC	8
Steenweg-de Graaff *et al.* (2017)	Netherlands	Not reported	Cohort study	Healthy pregnant women	5928	Plasma folate, preconception folic acid supplement use	Foetal head size, HC and growth	8
Timmermans *et al.* (2009)	Netherlands	2002-2006	Cohort study	Healthy pregnant women	6353	Periconceptional and early pregnancy folic acid supplement use	Foetal HC	8
Yusuf *et al.* (2019)	Florida	2010-2014	RCT	Healthy pregnant women who were current smokers	345	0.8 mg (control) or 4 mg (high-dose, intervention) folic acid supplement/day	Foetal HC, neonatal brain weight and BBR	8
Christian *et al.* (2003)	Nepal	1998-2001	RCT	Healthy pregnant women	4926	Folic acid (400 µg/day), folic acid-iron (60 mg ferrous fumarate), folic acid-iron-zinc (30 mg zinc sulphate), and multiple micronutrient supplement use	Neonatal HC	7
Nilsen *et al.* (2010)	Norway	2002-2003	Cohort study	Healthy pregnant women	2934	Plasma tHcy and folate, FFQ, folic acid supplement use	Neonatal HC	7
Timmermans *et al.* (2012)	Netherlands	2001-2006	Cohort study	Healthy pregnant women	3207	Plasma tHcy, serum vitamin B_{12}, plasma folate, FFQ, periconceptional folic acid supplement use	Foetal HC	7
Zou *et al.* (2020)	Netherlands	2002-2006	Cohort study	Healthy pregnant women	2095	Plasma folate, folic acid and multiple micronutrient supplement use	Foetal brain size	7
Bulloch *et al.* (2020)	Australia, New Zealand	2004-2011	Cohort study	Healthy pregnant women	5606	Intake of green leafy vegetables, folic acid supplement use	Neonatal HC	6
Catena *et al.* (2019)	Spain	2001-2003	RCT	Healthy pregnant women	85	5-m-THF supplement use	Neonatal HC	6
Husen *et al.* (2021)	Netherlands	Not reported	Cohort study	Healthy women from spontaneous or IVF/ICSI pregnancies	166	Self-reported questionnaire	Foetal brain structures (DTD, MTD, TTL and TTR)	6
Koning *et al.* (2015)	Netherlands	2009-2010	Cohort study	Healthy pregnant women	259	Serum and RBC folate, periconceptional folic acid supplement use	Foetal cerebellar growth	6

				(spontaneous and IVF/ICSI pregnancies)			(TCD, RCD and LCD)	
Lecorguillè *et al.* (2020)	France	2003-2006	Cohort study	Healthy pregnant women	1638	FFQ, dietary supplementation use	Neonatal HC	6
Parisi *et al.* (2018)	Netherlands	2013-2015	Cohort study	Healthy pregnant women (spontaneous and IVF/ICSI pregnancies)	126	Fasting plasma tHcy, serum vitamin B_{12}, serum and RBC folate, FFQ, periconceptional folic acid supplement use	Foetal cerebellum (TCD)	6
Tan *et al.* (2021)	Canada	2014-2016	Cohort study	Healthy pregnant women	709	Non-fasting serum tHcy and vitamin B_{12}	Neonatal HC	6
Brough *et al.* (2010)	UK	2002-2004	RCT	Healthy pregnant women	402	Multiple micronutrient supplement use, RBC folate	Neonatal HC	5
Koning *et al.* (2017)	Netherlands	2013-2015	Cohort study	Healthy pregnant women (spontaneous and IVF/ICSI pregnancies)	182	Folic acid supplement use	Foetal cerebellar growth (TCD, RCD and LCD)	5
Nakanishi *et al.* (2021)	Japan	2012-2014	Cohort study	Healthy pregnant women	124	Non-fasting plasma choline	Neonatal HC	5
Nemescu *et al.* (2020)	Romania	2018-2019	Cohort study	Healthy pregnant women	385	Folic acid supplement use	Foetal brain structures (MO distance and BPD)	5
Schlotz *et al.* (2010)	UK	Not reported	Cohort study	Healthy pregnant women	139	RBF folate, FFQ, folic acid supplement use	Neonatal HC	5
Gadgil *et al.* (2014)	India	2006-2008	Cross-sectional study	Healthy pregnant women	49	Plasma tHcy, vitamin B_{12} and folate, dietary recall, FFQ, pregnancy folic acid and vitamin B_{12} supplement use	Neonatal HC	4
Hossein-nezhad *et al.* (2011)	Iran	Not reported	Cohort study	Healthy pregnant women	113	Serum tHcy and folate, multivitamin supplement use, FFQ	Neonatal HC	4
Takimoto *et al.* (2011)	Japan	2007-2008	Cohort study	Healthy pregnant women	33	Non-fasting plasma tHcy, serum vitamin B_{12}, serum folate, vitamin B_6, dietary intake survey, vitamin supplement use	Neonatal HC	4
Jiang *et al.* (2016)	China	2015-2016	Cohort study	Healthy pregnant women	116	Serum tHcy, vitamin B_{12} and folate	Neonatal HC	3

Neumann *et al*. (2013)	Kenya	1984-1986	Cohort study	Healthy pregnant women	138	Quantitative weighing of food and dietary recall	Neonatal HC	3
Takimoto *et al*. (2007)	Japan	2001-2003	Cohort study	Healthy pregnant women	94	Non-fasting plasma tHcy, serum vitamin B_{12}, serum and RBC folate, vitamin B_6, dietary intake survey, vitamin supplement use	Neonatal HC	3

Abbreviations: brain-body weight ratio (BBR), biparietal diameter (BPD), diencephalon total diameter (DTD), food frequency questionnaire (FFQ), head circumference (HC), mesencephalon-to-occiput distance (MO), mesencephalon total diameter (MTD), randomized control trial (RCT), red blood cell (RBC), right cerebellar diameter (RCD), left cerebellar diameter (LCD), left and right telencephalon thickness (TTL and TTR), transcerebellar diameter (TCD), total homocysteine (tHcy).
